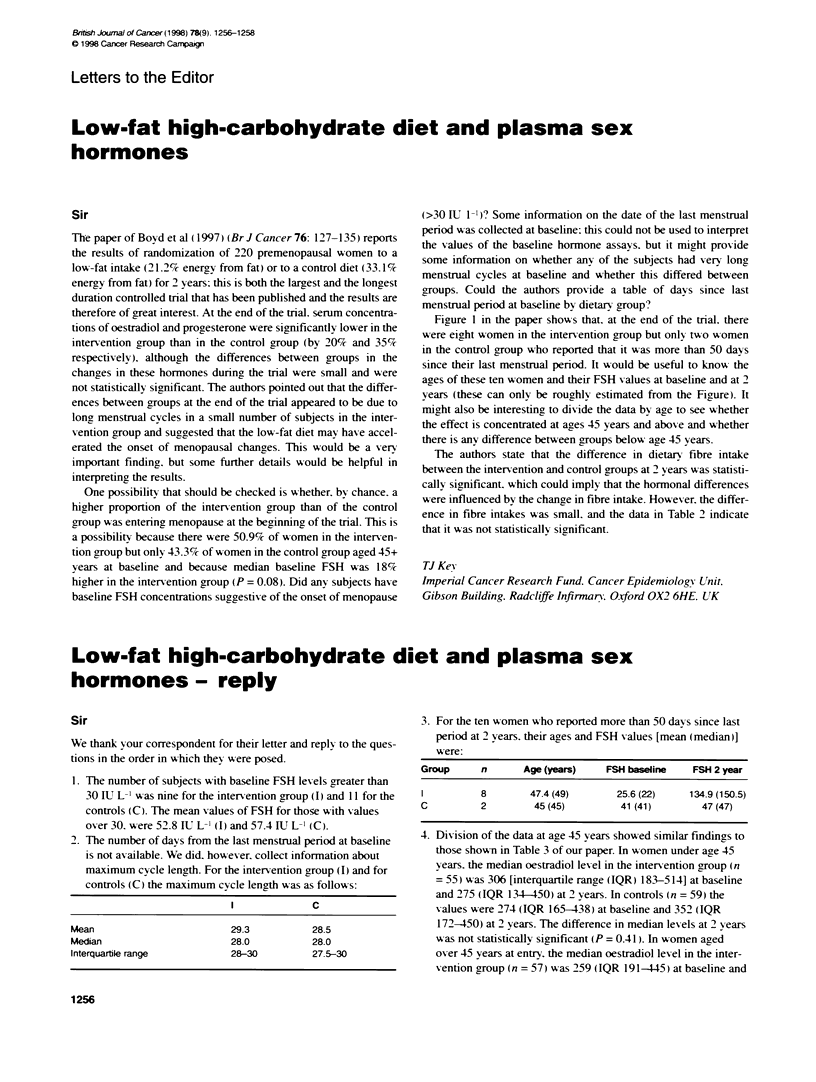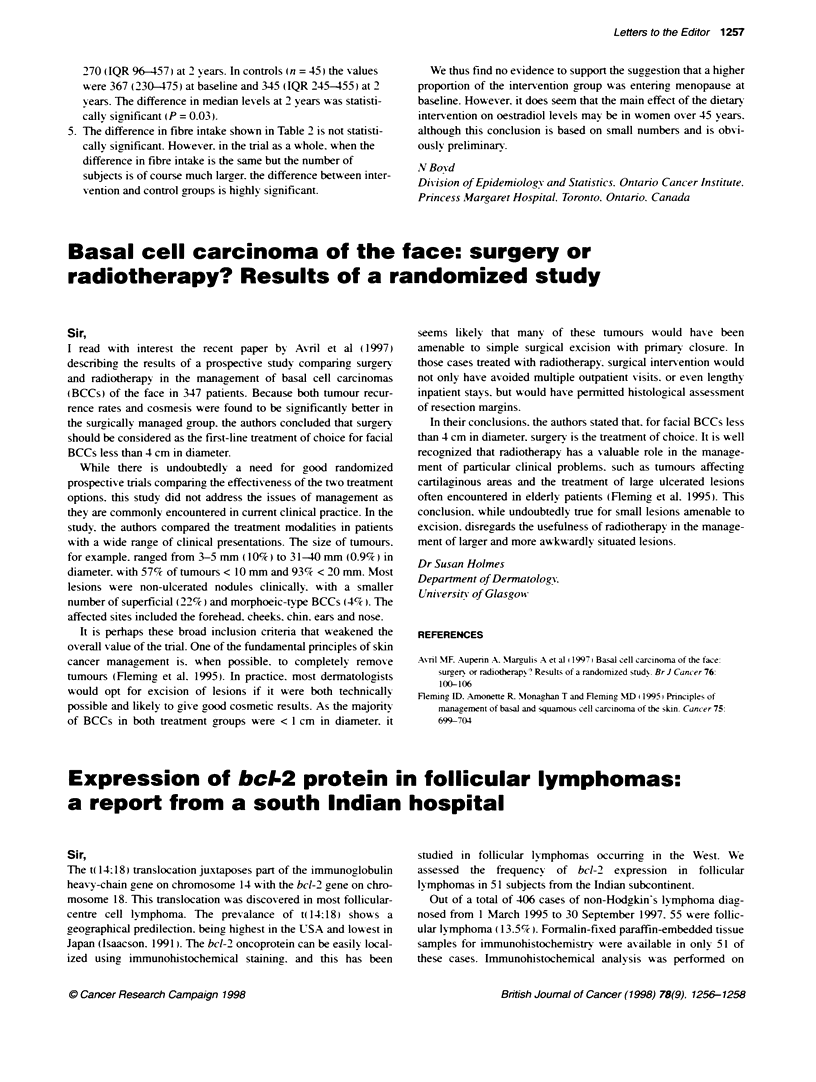# Low-fat high-carbohydrate diet and plasma sex hormones - reply

**Published:** 1998-11

**Authors:** N Boyd


Letters to the Editor

### Low-fat high-carbohydrate diet and plasma sex hormones

### Low-fat high-carbohydrate diet and plasma sex hormones – reply

### Sir

We thank your correspondent for their letter and reply to the questions in the order in which they were posed.

1. The number of subjects with baseline FSH levels greater than 30 IU $L^{-1}$ was nine for the intervention group (I) and 11 for the controls (C). The mean values of FSH for those with values over 30, were 52.8 IU $L^{-1}$ (I) and 57.4 IU $L^{-1}$ (C).
2. The number of days from the last menstrual period at baseline is not available. We did, however, collect information about maximum cycle length. For the intervention group (I) and for controls (C) the maximum cycle length was as follows:

| | I | C |
|---|---|---|
| Mean | 29.3 | 28.5 |
| Median | 28.0 | 28.0 |
| Interquartile range | 28–30 | 27.5–30 |

3. For the ten women who reported more than 50 days since last period at 2 years, their ages and FSH values [mean (median)] were:

| Group | n | Age (years) | FSH baseline | FSH 2 year |
|---|---|---|---|---|
| I | 8 | 47.4 (49) | 25.6 (22) | 134.9 (150.5) |
| C | 2 | 45 (45) | 41 (41) | 47 (47) |

4. Division of the data at age 45 years showed similar findings to those shown in Table 3 of our paper. In women under age 45 years, the median oestradiol level in the intervention group ($n$ = 55) was 306 [interquartile range (IQR) 183–514] at baseline and 275 (IQR 134–450) at 2 years. In controls ($n$ = 59) the values were 274 (IQR 165–438) at baseline and 352 (IQR 172–450) at 2 years. The difference in median levels at 2 years was not statistically significant ($P$ = 0.41). In women aged over 45 years at entry, the median oestradiol level in the intervention group ($n$ = 57) was 259 (IQR 191–445) at baseline and

270 (IQR 96–457) at 2 years. In controls ($n = 45$) the values were 367 (230–475) at baseline and 345 (IQR 245–455) at 2 years. The difference in median levels at 2 years was statistically significant ($P = 0.03$).

5. The difference in fibre intake shown in Table 2 is not statistically significant. However, in the trial as a whole, when the difference in fibre intake is the same but the number of subjects is of course much larger, the difference between intervention and control groups is highly significant.

We thus find no evidence to support the suggestion that a higher proportion of the intervention group was entering menopause at baseline. However, it does seem that the main effect of the dietary intervention on oestradiol levels may be in women over 45 years, although this conclusion is based on small numbers and is obviously preliminary.

*N Boyd*
*Division of Epidemiology and Statistics, Ontario Cancer Institute, Princess Margaret Hospital, Toronto, Ontario, Canada*